# Impact of a Whey Protein Hydrolysate Treated by Electrodialysis with Ultrafiltration Membrane on the Development of Metabolic Syndrome and the Modulation of Gut Microbiota in Mice

**DOI:** 10.3390/ijms241612968

**Published:** 2023-08-19

**Authors:** Valentine Renaud, Mélanie Faucher, Marie-Julie Dubois, Geneviève Pilon, Thibault Varin, André Marette, Laurent Bazinet

**Affiliations:** 1 Institute of Nutrition and Functional Food (INAF) and Department of Food Sciences, Pavillon Paul-Comtois, Université Laval, Québec, QC G1V 0A6, Canada; valentine.renaud.1@ulaval.ca (V.R.); melanie.faucher.2@ulaval.ca (M.F.); marie-julie.dubois@criucpq.ulaval.ca (M.-J.D.); genevieve.pilon@criucpq.ulaval.ca (G.P.); thibaut.varin.1@ulaval.ca (T.V.); andre.marette@criucpq.ulaval.ca (A.M.); 2 Laboratoire de Transformation Alimentaire et Procédés ElectroMembranaires (LTAPEM, Laboratory of Food Processing and ElectroMembrane Processes), Pavillon Paul Comtois, Université Laval, Québec, QC G1V 0A6, Canada; 3 Québec Heart and Lung Institute, Department of medicine, Université Laval, Québec, QC G1V 4G5, Canada

**Keywords:** whey protein hydrolysate, bioactive peptides, electromembrane extraction, electrodialysis with ultrafiltration membrane, digestion, gut microbiota

## Abstract

The development of Metabolic Syndrome (MetS) affects a large number of people around the world and represents a major issue in the field of health. Thus, it is important to implement new strategies to reduce its prevalence, and various approaches are currently under development. Recently, an eco-friendly technology named electrodialysis with ultrafiltration membrane (EDUF) was used successfully for the first time at a semi-industrial scale to produce three fractions concentrated in bioactive peptides (BPs) from an enzymatically hydrolyzed whey protein concentrate (WPC): the initial (F1), the final (F2) and the recovery fraction (F3), and it was demonstrated in vitro that F3 exhibited interesting DPP-IV inhibitory effects. Therefore, the present study aimed to evaluate the effect of each fraction on in vivo models of obesity. A daily dose of 312.5 mg/kg was administered to High Fat/High Sucrose diet (HFHS) induced C57BL6/J mice for eight weeks. The physiological parameters of each group and alterations of their gut microbiota by the fractions were assessed. Little effect of the different fractions was demonstrated on the physiological state of the mice, probably due to the digestion process of the BP content. However, there were changes in the gut microbiota composition and functions of mice treated with F3.

## 1. Introduction

Metabolic Syndrome (MetS) is a particular state of morbidity, characterized by the coexistence of several health disorders, such as hypertension, visceral obesity, dyslipidemia and impaired glucose homeostasis [1]. Several factors can contribute to the occurrence of MetS, including a sedentary lifestyle, genetic predispositions, or poor dietary habits [2]. This kind of health disorder can also lead to an elevated risk of developing type 2 diabetes (T2D) and cardiovascular diseases (CVD) [1]. Recent evidence suggested that the gut microbiota was implicated in the pathogenesis of the development of MetS factors. Indeed, the diversity of bacteria present in the intestine displays beneficial roles for the host as a nutrient, xenobiotic and drug metabolisms, maintenance of structural integrity of the epithelium, modulation of the immune system and protection against pathogens, as exampled by [3,4,5]. Induced obesity in mice and humans demonstrated major changes in terms of the composition and functions of their gut microbiota, leading to the development of disorders associated with the MetS [6,7]. Since the incidence of MetS is increasing worldwide and the complications generated after its development constitute a major issue in the field of health, strategies allowing the reduction of its prevalence have to be implemented [8].

Whey is a by-product of the dairy industry, produced in considerable amounts and containing a high concentration of proteins. When treated by enzymatic hydrolysis to produce whey protein hydrolysate (WPH), bioactive peptides (BPs) displaying many health effects can be released. The literature indicated that varying the enzymatic hydrolysis conditions (type of enzyme, duration of hydrolysis, etc.) could lead to the production of peptides with antihypertensive, antithrombotic, antioxidant and also anti-diabetic bioactivities [9,10]. Thus, the BP content of WPH may play a key role in the prevention and treatment of MetS due to their various health-promoting and disease risk-reducing agent effects. However, to increase the bioactivity of such hydrolysate, its purification or concentration is a necessary step.

Recently, our team worked on the separation of an enzymatically hydrolyzed commercially available whey protein concentrate (WPC, 35% protein on a dry basis) by electrodialysis with ultrafiltration membrane (EDUF), at a semi-industrial scale [11]. EDUF is part of a group of electrophoretic membrane processes, such as electromembrane extraction [12,13] or fixed boundary electrophoresis [14,15], which allow the separation of peptides according to their charges (electric field being the driving force) and size (due to the sieving effect of a porous membrane) [16,17,18,19]. This innovative and sustainable electrodialytic process, which has multiple membrane configurations, as well as numerous food and biopharmaceutical applications [17,20], allowed the production of three fractions: an initial whey protein hydrolysate (WPH), a final WPH after EDUF treatment and a peptide recovery fraction containing peptides migrated from the WPH during EDUF. The results obtained were promising since the treatment by EDUF allowed high migration, and the peptide recovery fraction exhibited up to a 4-fold higher in vitro inhibition of dipeptidyl-peptidase-IV (DPP-IV) than the initial WPH and, thus, displayed an anti-diabetic effect. Indeed, DPP-IV is a major factor in the degradation of endogenous incretin hormones: glucagon-like protein-1 (GLP-1) and glucose-dependent insulinotropic polypeptide (GIP). The role of these hormones is to stimulate insulin secretion and/or inhibit glucagon release in the presence of elevated glucose levels. Thus, inhibiting DPP-IV potentiates the action of incretins and reduces glucose levels [21].

In this context, the aim of this study was to evaluate in vivo the impact of these three fractions, obtained after EDUF separation at a semi-industrial scale, on MetS and gut microbiota. The specific objectives of the present study were to (1) evaluate the anti-diabetic effect of these different fractions in obese mice and (2) assess the effects of such fractions on the modulation of the gut microbiota.

## 2. Results

### 2.1. Physiological Effects of Fraction Supplementation

As expected, the High Fat/High Sucrose diet (HFHS) consumption induced a significant increase in mice’s total body weight gain, total energy intake, all fat depots and soleus weight as compared with those of the chow diet group (Table 1). However, none of the fractions had an impact on the physiological characteristics, except for the decrease in the weight of the pancreas in F1- and F3-treated groups.

### 2.2. Glucose Homeostasis and Insulin Sensitivity

**Figure 1:**
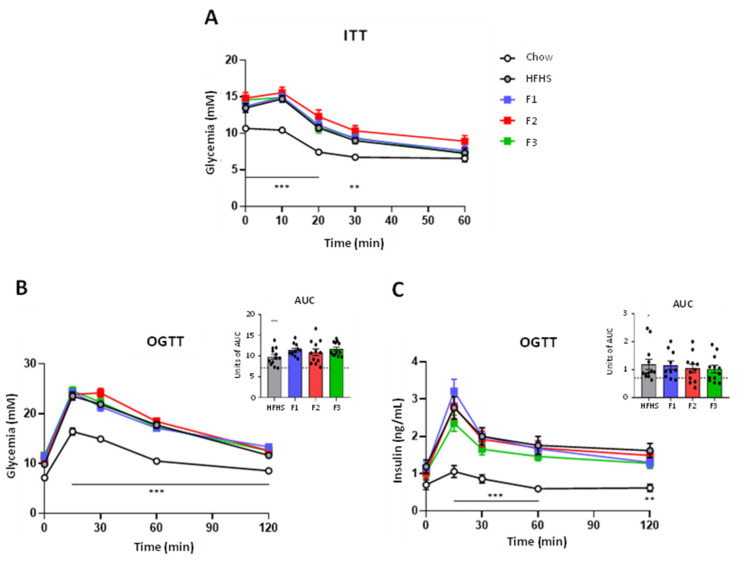
Impact of fraction supplementation in mice fed with HFHS diet on glucose homeostasis. Glycemic variation during ITT (**A**). Glycemic variation during OGTT (**B**). Insulinemic variation during OGGT (**C**). * *p* < 0.05 chow vs. HFHS, ** *p* < 0.01 chow vs. HFHS, *** *p* < 0.001 chow vs. HFHS.

After six weeks of dietary intervention, mice were subjected to an Insulin Tolerance Test (ITT) to assess the effects of fraction supplementation on HFHS diet-induced insulin resistance. As expected, HFHS-fed mice presented insulin resistance as compared to the chow diet-fed group. However, neither F1, F2, nor F3 reversed the insulin resistance induced by the HFHS diet (Figure 1A). In the eighth week of the experiment, an Oral Glucose Tolerance Test (OGTT) was also performed. Again, as expected, HFHS-fed mice were found to be glucose intolerant as compared to chow-fed animals, but neither fraction assessed were found to modulate the glycemic or the insulinemic response (Figure 1B,C).

### 2.3. Gut Microbiota Analyses

**Figure 2:**
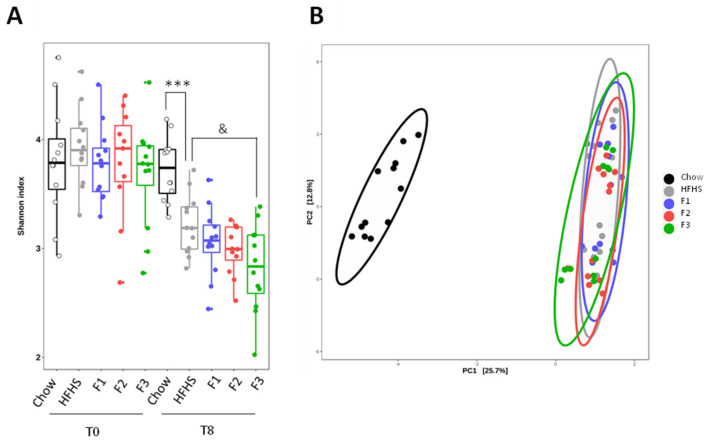
Effect of 8-week treatment with fraction supplementation to HFHS diet mice on alpha and beta diversity (*n* = 12). Alpha diversity was calculated with the Shannon index (**A**). Beta diversity was represented by principal component analysis (PCA) based on the Aitchison distance matrix (**B**). *** *p* < 0.001 chow vs. HFHS, & *p* < 0.05 HFHS vs. F3.

Stool samples were collected before and at the end of the experiment to evaluate the effect of fraction supplementation on the gut microbiota composition and functional pathways. The Shannon index was used to assess changes in the alpha diversity of each group (Figure 2A). After 8 weeks of treatment, a decrease in the Shannon index of all HFHS-treated groups was observed when compared to T0. Also, when compared to chow diet mice, the HFHS diet group showed a significant decrease in its alpha diversity. Supplementation with the different fractions did not reverse the loss of richness induced by the HFHS diet, and F3 worsened it, with a significant decrease in the Shannon index compared to the control HFHS diet group. Principal Component Analysis (PCA) was used to exhibit differences in the beta diversity of gut microbiota (Figure 2B). After the 8-week experiment, chow and all HFHS diet groups clustered differentially. F1-, F2- and F3-treated mice clustered similarly to the HFHS diet group.

**Figure 3:**
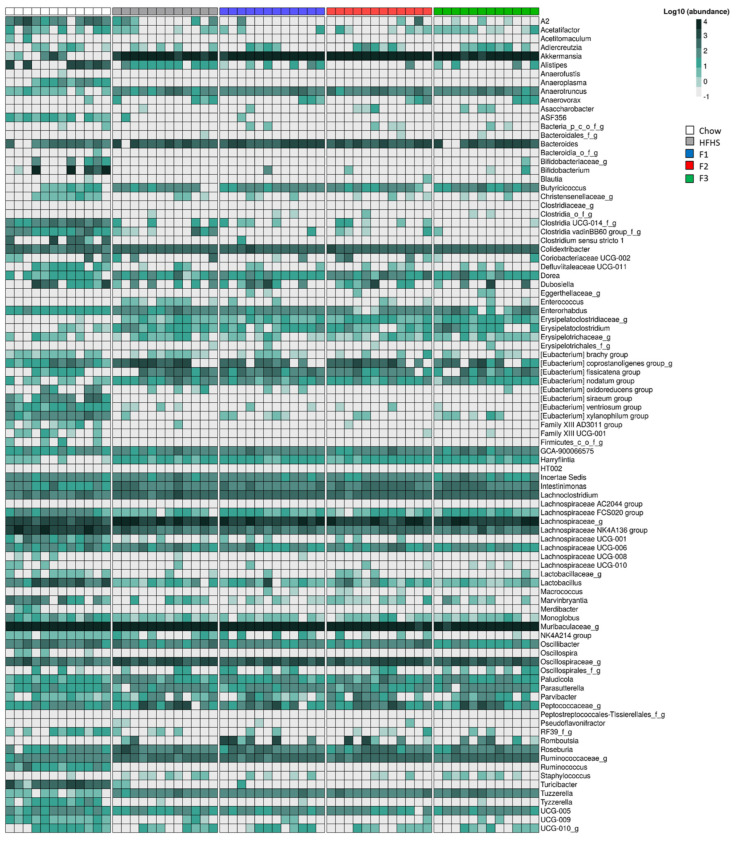
The bacterial (log10-transformed) abundance of the microbiota community after 8 weeks of treatment represented by a heatmap. The presence of ‘p,’ ‘c,’ ‘o,’ ‘f,’ and ‘g’ at the end of the taxon denotes an unclassified phylum, class, order, family, and genus, respectively.

Bacterial abundance was then represented using a heatmap at the genus level (Figure 3). Data indicated a change in the bacterial abundance between chow and HFHS diet groups. Indeed, the HFHS diet induced an increase of numerous bacterial genera, such as *Akkermansia, Bacteroides*, *Erysipelatoclostridaceae, Eubacterium corprostanoligenes*, *Oscillospiraceae* and *Ruminococcaceae,* and a decrease in *Anaeroplasma*, multiple *Eubacterium* species, *Lachnospiraceae_UCG001*, *Tyzzerella* or *UCG_009*. The same observations were observed when comparing the bacterial abundance between the HFHS diet group and the fraction-treated groups.

**Figure 4:**
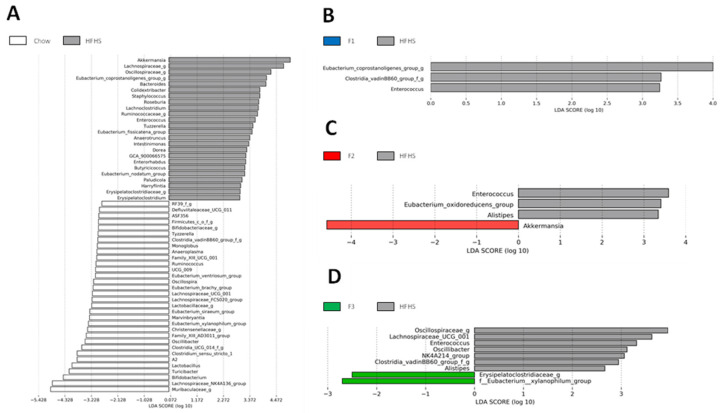
Linear discriminant analysis (LDA) combined with linear effect size measurements (LEfSe) was used to explore the taxa that enable discrimination between (**A**) chow vs. HFHS, (**B**) HFHS vs. F1, (**C**) HFHS vs. F2 and (**D**) HFHS vs. F3.

Next, differential bacterial abundance between groups at the genus level was determined using Linear Effect Size (LEfSe) (Figure 4). As expected, after 8 weeks of HFHS-induced diet, significant differences in bacterial composition were observed between HFHS mice and the chow diet group (Figure 4A). The gut microbial communities of F1-treated mice were discriminated from those of the HFHS-fed group by decreases in abundance of *Eubacterium coprostanoligenes, Clostrida vadin BB60* and *Enterococcus* (Figure 4B). Administration of F2 to HFHS mice was associated with an increased abundance of *Akkermansia* and lower representations of *Enterococcus, Eubacterium oxidoreducens* and *Alistipes* (Figure 4C). Increased abundances of *Erysipelatoclostridiaceae* and *Eubacterium xylanophylum* were found in the F3-treated group compared to HFHS diet mice, whereas *Oscilospiraceae*, *Lachnospirales UCG_001, Enterococcus, Oscillibacter, NK4A214, Clostridia vadin BB60* and *Alistipes* were found in lower abundance (Figure 4D).

**Figure 5:**
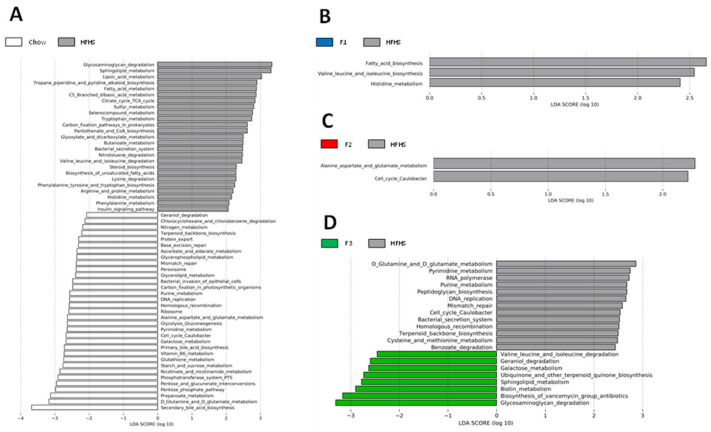
Prediction of the functional pathways from 16S gene-based data performed using PICRUSt2. LDA scores were used for differentially abundant functional pathways between (**A**) chow vs. HFHS, (**B**) HFHS vs. F1, (**C**) HFHS vs. F2 and (**D**) HFHS vs. F3.

Finally, the PICRUSt 2 pipeline was applied to assess functional alterations induced by the fraction supplementation in the gut microbiome of mice (Figure 5). As expected, the obesity-driven diet-induced alterations in metabolic pathways were exhibited by the HFHS diet group compared to the chow group (Figure 5A). Administration of F1 to HFHS diet mice was associated with a decrease in functional pathways linked to fatty acid and amino acid (AAs) metabolism (Figure 5B). Microbial pathways assigned to AAs metabolism and cellular process were decreased in F2-treated mice compared to the HFHS diet group (Figure 5C). F3-treated mice exhibited a decrease in functions implicated in various metabolism pathways (e.g., AAs, nucleotides, terpenoids and polyketides and xenobiotics), DNA replication and repair processes and cellular processes compared to HFHS mice. Also, an overrepresentation of functions associated with other multiple metabolism pathways (e.g., AAs, terpenoids and polyketides, carbohydrates, cofactors and vitamins and lipids) and glycan biosynthesis were observed in the F3-treated group compared to HFHS diet mice (Figure 5D).

## 3. Discussion

The aim of this study was to evaluate, in vivo, the anti-diabetic effect(s) of the different fractions produced by Faucher et al. 2022 using EDUF in obese mice and to assess the effects of such fractions on the modulation of the gut microbiota [11]. The overall results indicated that none of the fractions impacted the mice’s physiological parameters except the pancreas weight. Indeed, no variation was observed in total energy intake, total body weight and fat mass in mice treated with one of the fractions produced by EDUF. Concerning the organ weight, only the pancreas of F1- and F3-treated groups were lighter than the HFHS diet group. The literature indicated that whey protein or protein hydrolysate could stimulate the secretion of cholecystokinin, a satiety hormone decreasing gastric emptying and food intake and stimulating pancreatic secretion [22,23,24]. Interestingly, some studies indicated that the elevated presence of branched-chain AAs (BCAAs) such as leucine, isoleucine and valine seemed to be responsible for the secretion of this hormone and other regulatory hormones such as GLP-1 and insulin, and in turn, could inhibit satiety and food intake [25,26]. When assessing glucose homeostasis and insulin sensitivity, no change was observed in F1, F2 and F3-treated mice compared to the HFHS diet group. 

**Figure 6:**
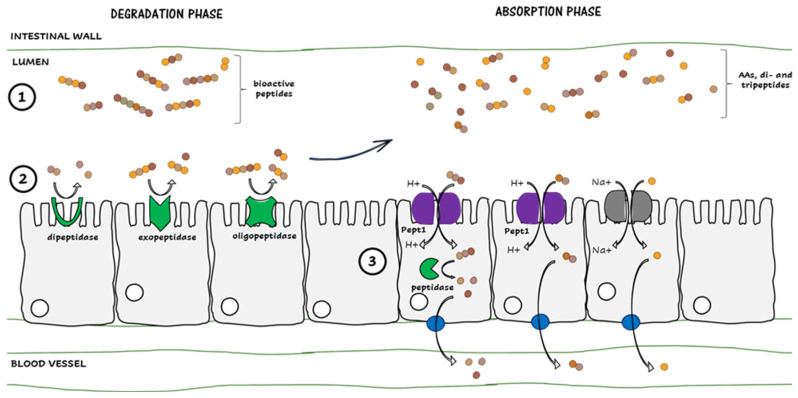
Schematic representation of the digestion process of BPs. Step 1. Arrival of the BPs in the intestinal lumen. Step 2. Degradation of the BPs by peptidases. Step 3. Absorption of the metabolites through the epithelia to blood vessels.

A way to explain these results resides in the digestion process of the fractions and, more particularly, in the BP content degradation and transport in the bloodstream (Figure 6). When ingested, BPs travel from the stomach, where they undergo a thin degradation, to the intestine (Figure 6. step 1). Once in the intestinal lumen, the BPcontent undergoes an extensive degradation by different types of peptidases present on the apical side of the epithelial, such as dipeptidases, which break dipeptides in free AAs, exopeptidases (A, P, etc.) which cleave particular N-terminal AAs and endopeptidases breaking peptides in the middle of their sequence [27] (Figure 6, step 2). This degradation process is necessary for the absorption phase since only free AAs, di- and tripeptides can pass through the epithelia using two well-known transporters: Pept1 and a Na^+^-dependent pump [28] (Figure 6. step 3). Once in the cell, some di- and tripeptides can undergo further degradation by internal peptidases before being released in the stream [28,29]. When analyzing the number of AAs composing each sequence of the BPs content of our fractions, it was observed that most of the peptides were composed of more than three AAs, and it was possible that they were further degraded during the digestion process (Table S1). Since a loss in the structure of BPs is associated with a loss of bioactivity, as already observed by Dlask et al. 2018 [20], it could be hypothesized that our fractions also lost their bioactivity, explaining the fact that there was no change in the physiological parameters and the glucose homeostasis of the treated mice. Also, these results confirmed that some discordances can appear between in vitro and in vivo assays, as already reported by Geerlings et al. 2006 and Mirzapour-Kouhdasht et al. 2022 [30,31], as well as for in silico and in vivo analyses of tryptic WPH [32]. Furthermore, amongst the 19 peptides identified in the fractions, 18 contained at least one BCAA in their sequence, which could be released during the digestion process. This was interesting since some studies indicated changes in glucose tolerance, insulin sensitivity or glucose uptake in different models where pure BCAAs were administered at superior doses compared to our study [33,34,35,36,37]. This indicated that the content in BCAAs of our fractions could have been too low to induce significant changes at the physiological stage in our model.

Regarding the digestion process, again, the metabolites liberated during the degradation phase remained in the lumen prior to absorption and were probably in contact with the gut microbiota. Therefore, the effect of these metabolites on the modulation of the composition and the functions of the gut microbiota was investigated. Regarding the results of diversity, there was only a loss in the alpha diversity of the F3-treated group compared to the HFHS diet mice but no change in the beta diversity. Furthermore, the heatmap indicated changes in the bacterial abundance of the HFHS-treated mice compared to the chow group, but no major change was found when comparing F1-, F2- and F3-treated groups and the HFHS diet mice. As expected, the LEfSe analysis indicated major changes in the microbial community and the metabolic pathways exhibited by the gut microbiota of the HFHS diet group compared to the chow diet mice. As already deeply reviewed, diet-induced obesity leads to bacterial dysbiosis associated with changes in the functions of the gut microbiota since these functions are directly associated with the type of bacteria present in the intestinal [38]. When the comparison of the fraction-treated groups vs. the HFHS diet group was analyzed, the LEfSe analysis revealed that all fraction-treated groups were associated with a reduction in the genera *Enterococcus*. Many different strains of *Enterococcus* spp. exist, including both pathogenic and commensal microorganisms; some are antibiotic-resistant, responsible for nosocomial infections or capable of producing bacteriocins and inhibiting the growth of pathogens [39,40]. Therefore, their implication in health is mitigated, and their decreased abundance due to the fraction treatments could be further investigated to determine if this decrease had a positive or negative effect on the host. Furthermore, when supplemented with fractions F2 and F3, mice presented a reduction in the genera *Alistipes abundance*. A decrease in the abundance of this type of bacteria is associated with a protective effect in DSS-colitis-induced mice, while an increase in the genera is associated with dysfunction of the intestinal barrier [41,42]. Thus, the decrease in *Alistipes* abundance in F2- and F3-treated mice could be associated with a beneficial effect of the fraction in mice. Interestingly, after an 8-week supplementation with F2, mice presented an increased abundance of *Akkermansia*, a beneficial bacterium for the host, when compared to the HFHS diet group. Finally, F3-treated mice presented an increase in the abundance of *E. xylanophylum* when F1- and F2-treated mice induced a decrease in *E. coprostanoligenes* and *E. oxidoreducens*. The bacteria *E. xylanophylum* is a potent butyrate producer, which is associated with beneficial effects on health since short-chain fatty acids (SCFAs) are a source of energy for epithelial cells and contributeto the homeostasis of the intestinal environment [43,44].

Concerning the metabolic pathways exhibited by the gut microbiota, only F3 induced major alterations in the gut microbiota of mice. Indeed, F3-treated mice presented an increase in abundance of the metabolic pathway of valine, leucine, and isoleucine degradation. As explained before, the BCAAs content of the BPs present in the fraction F3 would have been released during the degradation phase of the digestion and the fact they remain in the lumen could have induced their degradation by the gut microbiota of mice. This would explain the increase in abundance of this metabolic pathway. Furthermore, there was an increase in the pathway of galactose metabolism that could be explained by the fact that F3 contained more lactose than the other fractions (26% more than F1). Lactose is a sugar, and when degraded in the gut, it releases one molecule of glucose and one molecule of galactose. Then, the release of galactose could have induced degradation by commensals, leading to the production of pyruvate, a source of energy for bacteria such as the *Eubacterium* type. This could explain the increased abundance of *E. xylanophylum* in the gut microbiota of F3-treated mice and the decreased abundances of *E. coprostanoligenes* and *E. oxidoreducens* in F1- and F2-treated mice, respectively, which had a depleted source of lactose compared to F3. Finally, the biosynthesis of the vancomycin pathway was increased in the F3-supplemented group compared to the HFHS diet mice. Vancomycin belongs to a group of antibiotics that inhibits the growth of *Enterococcus*, and the increased abundance of this pathway could explain the decreased abundance of this bacteria in the F3-treated mice.

## 4. Materials and Methods

### 4.1. Supplementation Samples

Three WPC-based treatments were used as oral supplementation. Briefly, a commercial WPC 35 (35% protein on a dry basis) provided by Lactalis (Victoriaville, QC, Canada) was hydrolyzed with a commercial trypsin IV containing 11% of chymotrypsin (Neova Technologies Inc., Abbotsford, BC, Canada) (1:200 enzyme-to-substrate ratio) for 4 h at 37 °C in accordance with previous studies [45,46]. The resulting WPH was then treated for 6 h by EDUF under a constant current of 1.2 A, according to [11,46,47]. Briefly, the EDUF cell was composed of repeating units, themselves composed of two different cells (C1 and C2), allowing the positively charged peptides to migrate from the WPH (C1) to the peptide recovery fraction (C2) through a 50 kDa molecular weight cut-off UF membrane (see Supplementary Material for more information). This specific UF membrane was selected, in accordance with previous studies reported in the literature, due to its high recovery of peptides of interest [45,48], such as peptides having strong bioactivities, while limiting the migration of negatively charged peptides (selectivity) [45,48] and fouling by peptides [49,50]. F1 corresponded to the initial WPH, F2 to the final WPH obtained after EDUF and F3 to the peptide recovery fraction containing peptides migrated during EDUF. The final composition of each fraction is listed in Table 2, and the BP content is listed in Supplementary Material (Table S1). All three fractions were then demineralized up to 90% by conventional electrodialysis (ED) under a constant current of 9V [11,51,52]. The ED cell configuration was made of alternating cation and anion-exchange membranes (Astom, Tokyo, Japan), allowing the formation of the electrode rinsing solution, concentrate and diluate compartments. The fractions were demineralized by circulating in the diluate compartment (see Supplementary Material for more information). The detailed production protocol of the treatment supplementation is described in Faucher et al. 2022 [11]. After demineralization, the demineralized initial WPH (F1), demineralized peptide recovery fraction (F3), and demineralized final WPH (F2) were recovered and freeze-dried.

### 4.2. Animals and Dietary Treatments

Sixty C57BL/6J male mice (six-week-old) were received from The Jackson Laboratory (Bar Harbor, ME, USA) and single-housed in ventilated cages in a controlled environment (12 h light/dark cycle at 25 °C) with ad libitum access to food and water. After two weeks of acclimation on a standard chow diet (Teklad diet 2018), mice were randomly divided into five groups (*n* = 12) as follows: one control chow diet group (Chow, 3.1 kcal/g), one control HFHS diet group (HFHS, 5.49 kcal/g) and three fraction-treated HFHS diet groups. The composition of the HFHS diet is referenced in Anhê et al. 2015 [53]. Each group received a daily oral gavage of the vehicle (water) for both control groups and one of the fractions produced (F1, F2 or F3) for the treated groups (312.5mg/kg). The daily dose received by each group was chosen according to previous experiments where it was estimated that this dose corresponded to 1.5g/d for a 60kg human [54,55]. Also, it was calculated for a reasonable daily consumption for humans (3 pills per day). During the eight-week-experiment, body weight was assessed twice a week, and food intake was assessed three times a week. Before treatment (T0) and at week eight (T8), fresh feces were collected and stored at −80 °C until further analysis, and body composition was assessed by nuclear magnetic resonance using Bruker’s Minispec LF90II (Bruker Optics, Milton, ON, Canada). At the end of the experiment, mice were anesthetized by isoflurane inhalation and euthanized by cardiac puncture and cervical dislocation. Tissue and organs were collected, weighed, and stored at −80 °C until further experiments. All procedures were approved (#2021-741) by the Université Laval Animal Ethics Committee and followed the guidelines for the care and use of laboratory animals.

### 4.3. Insulin Tolerance Test

After six weeks of treatment (T6), an ITT was conducted on mice. Animals fasted for 6 h, and glycemia was measured in blood (30 µL) drawn from the caudal vein (0 min) with a One Touch Verio Flex glucometer (LifeScan Canada, Burnaby, BC, Canada). Then, insulin was injected intraperitoneally, and glycemia was measured after 10, 20, 30 and 60 min.

### 4.4. Oral Glucose Tolerance Test

Four days before the sacrifice at week eight, an Oral Glucose Tolerance Test (OGTT) was assessed on mice. Animals fasted overnight for 12 h, and blood (30 µL) was drawn from the caudal vein (0 min) to measure glycemia and insulinemia. Then, mice orally received a solution of dextrose (1 g/kg of body weight) and blood samples were collected after 15, 30, 60 and 120 min. Glycemia was measured using a One Touch Verio Flex glucometer (LifeScan Canada, Burnaby, BC, Canada), and insulinemia was measured using a mouse ultrasensitive insulin ELISA kit according to the manufacturer’s instruction (Alpco, Salem, MA, USA).

### 4.5. Fecal Sample Processing and 16S rRNA Gene-Based Sequencing

Fresh fecal samples were collected on T0 and T8 and immediately stored at −80 °C. As previously described, DNA was extracted, and the construction and sequencing of 16S rRNA gene-based libraries of the fecal microbiota were carried out [56]. High-throughput sequencing was performed at the Centre d’Expertise et des Services Génome (Quebec, QC, Canada).

### 4.6. Gut Microbiota Analyses

Forward and reverse primers were removed from raw paired-end reads using Cutadapt (v3.1) [57]. Sequences were then demultiplexed, denoised, dereplicated and merged with the DADA2 package (v1.16) in the R software (v4.0.0) [58]. The resulting amplicon sequence variants (ASVs) table was assigned taxonomy using the Ribosomal Database Project RDP classifier algorithm (v2.2) [59], trained against the SILVA database 138.1 [60]. Taxa that appeared less than three times in the entire dataset were removed. Samples were rarefied to an even sampling depth of 16,971 sequences per sample.

### 4.7. Functional Prediction of Gut Bacterial Communities

Prediction of functional genes was performed using KEGG (Kyoto Encyclopedia of Genes and Genomes) and PICRUSt2 (Phylogenetic Investigation of Communities by Reconstruction of Unobserved States) [61]. Briefly, the ASVs table was used to predict the KEGG pathway abundances using picrust2_pipeline.py as provided (https://github.com/picrust/picrust2/wiki/Full-pipeline-script, accessed on 5 March 2023) [62].

### 4.8. Statistical Analyses

Statistical analyses were performed using Graphpad prism software (v8.0, Graphpad Software Inc., San Diego, CA, USA). One-way ANOVA with either a post hoc Student’s *t*-test or a post hoc Dunnett’s test was used to assign significance to the comparisons between the chow diet group vs. HFHS diet group and HFHS diet group vs. HFHS diet treated groups, respectively. Time points within distinct groups were compared using two-way repeated measures ANOVA. Data are expressed as mean ± SEM. All results were considered statistically significant at *p* < 0.05.

Regarding gut microbiota data, the Shannon index, which reflects the richness and evenness of microbial representation in a sample, was used to analyze the alpha diversity of samples. The similarity of microbial communities between samples was calculated using PCA based on Aitchison distance. The detection of differentially abundant taxa or pathways between groups was performed with the metagenomics biomarker discovery tool for LEfSe using an LDA score threshold ≥2.5 or ≥2.0, respectively [63].

## 5. Conclusions

The results of our study indicated that the regular consumption of the three fractions produced by EDUF did not induce physiological alterations in mice, such as improvement of obesity and glucose homeostasis. These results were explained by the digestion process that limited the absorption of intact BPs, thus decreasing their anti-diabetic properties. However, the regular consumption of the peptide recovery fraction (F3) induced changes in the composition of the gut microbiota and its expressed metabolic pathways, but these alterations could not be translated into physiological effects. Thus, the modulation of the gut microbiota could not decrease the risk factor for the development of the MetS in our model. It would be interesting to increase the dose and/or the duration of the experiment in order to determine if alterations could be observed at the physiological stage. Furthermore, it could be interesting to evaluate different parameters of enzymatic hydrolysis in terms of time or enzymes used, to produce a WPH containing smaller BPs. Thus, the reduced size of the BP sequences could be less degraded during the degradation process in the intestine and could be absorbed almost intact in the bloodstream. Also, using a WPC 55 or 80 (55 or 80% of protein on a dry basis), other whey protein concentrates industrially available, instead of a WPC 35 (35% of protein on a dry basis) to produce the initial WPH could allow the production of fractions with lower lactose contents. Consequently, the bioactivity of these fractions would be more related to the presence of BPs. Finally, it could be interesting to determine the extent of the degradation of the BP content and to assess the rate absorption of the degradation products via Caco-2 cell culture.

Finally, since whey is a by-product of the dairy industry, and produced in large amounts each year (9 kg of whey produced per kg of cheese), its valorization by EDUF treatment, a sustainable and eco-friendly process [17] is of great interest and relevance in terms of human health but also for the environment in the framework of a circular economy [19].

## Figures and Tables

**Table 1:** Effect of fraction supplementation on mice physiology.

	Chow	HFHS	F1	F2	F3
Total weight gain (g)	4.24 ± 0.40 ***	9.98 ± 0.92	10.56 ± 1.05	11.61 ± 0.96	10.22 ± 0.96
Total energy intake (kcal)	602.59 ± 14.41 **	693.25 ± 20.28	669.05 ± 16.71	708.97 ± 15.00	665.93 ± 11.24
Visceral fat pad (g)	1.16 ± 0.11 ***	3.40 ± 0.32	3.38 ± 0.39	3.83 ± 0.37	3.45 ± 0.28
Subcutaneous fat pad (g)	0.34 ± 0.03 ***	0.80 ± 0.07	0.90 ± 0.08	0.99 ± 0.14	0.89 ± 0.09
Brown adipose tissue (g)	0.08 ± 0.01 ***	0.12 ± 0.01	0.12 ± 0.01	0.13 ± 0.01	0.12 ± 0.01
Total lean mass (g)	19.71 ± 0.49	20.3 ± 0.33	20.18 ± 0.33	20.58 ± 0.35	19.75 ± 0.33
Total fat mass (g)	2.73 ± 0.31 ***	7.28 ± 0.64	7.77 ± 0.76	8.29 ± 0.83	7.71 ± 0.56
Gastrocnemius (g)	0.26 ± 0.01	0.26 ± 0.00	0.26 ± 0.00	0.27 ± 0.01	0.25 ± 0.00
Soleus (g)	0.02 ± 0.00 *	0.02 ± 0.00	0.02 ± 0.00	0.02 ± 0.00	0.02 ± 0.00
Brain (g)	0.43 ± 0.01	0.43 ± 0.00	0.43 ± 0.00	0.43 ± 0.01	0.42 ± 0.00
Heart (g)	0.14 ± 0.00	0.13 ± 0.00	0.13 ± 0.00	0.13 ± 0.00	0.14 ± 0.00
Kidneys (g)	0.33 ± 0.01	0.35 ± 0.01	0.34 ± 0.00	0.35 ± 0.01	0.33 ± 0.01
Liver (g)	1.06 ± 0.04	0.99 ± 0.06	1.09 ± 0.04	1.12 ± 0.06	1.06 ± 0.03
Pancreas (g)	0.29 ± 0.01	0.32 ± 0.03	0.26 ± 0.01 #	0.31 ± 0.02	0.27 ± 0.01 &

Mean ± SEM, * *p* < 0.05 chow vs. HFHS, ** *p* < 0.01 chow vs. HFHS, *** *p* < 0.001 chow vs. HFHS, # *p* < 0.05 HFHS vs. F1, and & *p* < 0.05 HFHS vs. F3. HFHS: High-Fat High-Sucrose, SEM: standard error of the mean.

**Table 2:** Proximal composition of each fraction produced.

	F1	F2	F3
Proteins (g/100 g on a dry basis)	38.40 ± 0.21	40.54 ± 0.19	19.56 ± 0.03
Ash content (g/100 g on a dry basis)	0.89 ± 0.03	0.87 ± 0.03	1.02 ± 0.03
Calcium	0.11 ± 0.01	0.11 ± 0.01	0.08 ± 0.00
Potassium	0.02 ± 0.00	0.03 ± 0.00	0.02 ± 0.01
Magnesium	0.02 ± 0.00	0.03 ± 0.00	0.02 ± 0.00
Phosphorus	0.06 ± 0.01	0.09 ± 0.01	0.22 ± 0.02
Sodium	0.21 ± 0.01	0.22 ± 0.01	0.11 ± 0.00
Lactose (g/100 g on a dry basis)	44.94 ± 0.92	39.52 ± 0.43	68.76 ± 1.96
Moisture content (g/100 g on a dry basis)	1.07 ± 0.00	5.42 ± 0.11	2.51 ± 0.19

## Data Availability

Data is contained within the article.

## Supplementary Materials

The following supporting information can be downloaded at: https://www.mdpi.com/article/10.3390/ijms241612968/s1. References [11,31,32,63,64,65] are cited in the supplementary materials.
