# Supplementary material for: Impact of a Whey Protein Hydrolysate Treated by Electrodialysis with Ultrafiltration Membrane on the Development of Metabolic Syndrome and the Modulation of Gut Microbiota in Mice
Source: Int J Mol Sci. 2023 Aug 19;24(16):12968. doi: 10.3390/ijms241612968 (PMC10454911; doi:10.3390/ijms241612968)
Supplement: Supplementary file 1 [file ijms-24-12968-s001.zip › ijms-2556854-supplementary.pdf]

Table S1. Major peptides recovered in F3, characteristics, associated bioactivities and relative abundance (in %) versus initial content in F1 (in %) (adapted from [11,31,32,63-65).

Peptide sequence	Observed mass (Da)	Retention time (min)	Net charge at pH7	Peptide source	Associated bioactivities	Relative abundance in F3	Relative abundance in F1
VY	280.14	2.5	0	β-lg (41-42)	ACE inhibitor	3.75±1.25	2.43±0.48
ILDK<br>+AEK<br>+ IIAEK	487.30<br>346.15<br>572.35	2.8	0<br>0<br>0	α–la (95-98)<br>β-lg (73-75)<br>β-lg (71-75)	Calcium binding<br>ACE inhibitor<br>ACE inhibitor, DPP-IV inhibitor, Hypocholesterolemic	4.95±0.41	4.52±0.80
DAQSAPLR	856.44	4.04	0	β-lg (33-40)	ACE inhibitor	3.53±0.26	3.43±0.19
IVTQTMK	819.45	4.4	1	β-lg (2-8)	DPP-IV inhibitor	1.41±0.11	1.14±0.06
IDALNENK	915.47	5.1	-1	β-lg (84-91)	ACE inhibitor, DPP-IV inhibitor, Hypocholesterolemic	0.45±0.00	1.43±0.03
GLDIQK	672.38	5.6	0	β-lg (9-14)	ACE inhibitor	3.08±0.06	3.50±0.04
ALPMHIR<br>+PMHIR	836.47<br>656.35	7.91	1.1<br>1.1	β-lg (142-148)<br>β-lg (144-148)	ACE inhibitor, Antioxidant<br>ACE inhibitor	1.66±0.14	0.22±0.00
LIVTQTMK	932.54	8.26	1	β-lg (1-8)	DPP-IV inhibitor	5.55±0.28	3.88±0.11
ALPM<br>+VGINY	430.22<br>564.29	8.63	0<br>0	β-lg (142-145)<br>α–la (99-103)	ACE inhibitor<br>Non-reported	5.41±0.08	3.33±0.42
VLVLDTDYKK	1192.67	9.32	0	β-lg (92-101)	DPP-IV inhibitor	0.80±0.05	3.14±0.53
VAGTWY	695.33	10.62	0	β-lg (15-20)	ACE inhibitor, DPP-IV inhibitor, Antioxidant-ORAC	28.04±0.65	13.26±0.35
VLVLDTDYK	1064.57	11.25	-1	β-lg (92-100)	ACE inhibitor	1.02±0.17	2.55±0.31
TKIPAVF	774.46	12.27	1	β-lg (76-82)	Antibacterial	5.94±0.36	2.61±0.23
IPAVF	545.32	13.37	0	β-lg (78-82)	ACE inhibitor, DPP-IV inhibitor	10.79±0.17	3.78±0.13
YLLF	554.31	17.08	0	β-lg (102-105)	ACE inhibitor	3.77±0.23	1.98±0.28

β-lg : β-lactoglobulin, α-la: α-lactalbumin, WPH: whey protein hydrolysate

Electrodialytic Cells

1) *Electrodialysis with ultrafiltration membranes for peptide recovery*

The EDUF experiments were carried out using an industrial unit fabricated by Eurodia Industrie SAS (Pertuis, France), with an effective surface of 560 cm^2 and configured to recover positively charged peptides. The cell configuration used was first developed by Goeffroy et al. 2022 [46] to overcome some technological limitations. Thereby, as described in detail by Geoffroy et al. 2022 [46], the cell was composed of cation-exchange membranes (CEMs) (Astom, Tokyo, Japan), anion-exchange membranes (AEMs) (Astom, Tokyo, Japan) and UF membranes (Synder, Vacaville, CA, USA) stacked between two dimensionally stable electrodes (DSE).

2) Conventional electrodialysis for demineralization

The initial WPH (F1), final WPH (F2) and peptide recovery fraction (F3) were demineralized using a MP type cell (ElectroCell AB, Täby, Sweden). The anode was a dimensionally stable electrode (DSA-O_2), and the cathode was a food-grade stainless steel electrode. During treatment, all solutions were circulating through three closed loops connected to reservoirs, pumps (Baldor Electric Company, Fort Smith, AR, USA) and flowmeters (Blue-White Industries Ltd., San Diego, CA, USA) [11].
